# Amisulpride Augmentation for Clozapine-Refractory Positive Symptoms: Additional Benefit in Reducing Hypersialorrhea

**DOI:** 10.1155/2015/408179

**Published:** 2015-03-09

**Authors:** Fabiani Bogorni, Frederico Fernandes Moreira, Eduardo Mylius Pimentel, Géder Evandro Motta Grohs, Alexandre Paim Diaz

**Affiliations:** ^1^Programa de Residência Médica em Psiquiatria do Instituto de Psiquiatria de Santa Catarina (IPQ/SC), 88.123-300 São José, SC, Brazil; ^2^Centro de Neurociências Aplicadas, Universidade Federal de Santa Catarina, 88.040-970 Florianópolis, SC, Brazil

## Abstract

One-third to half of patients taking clozapine suffer from refractory symptoms despite adequate treatment. Among other adverse effects, clozapine-induced hypersalivation (CIH) occurs in approximately half of all patients. This is a case of a 30-year-old male with refractory schizophrenia; in this patient, the remission of residual positive symptoms, as well as the reduction of CIH, was achieved by treatment with clozapine augmented with amisulpride.

## 1. Introduction

Approximately 30% of patients diagnosed with schizophrenia are resistant to antipsychotics and are candidates for treatment with clozapine. Still, one-third to half of patients taking clozapine suffer from refractory symptoms despite adequate treatment [1, 2]. Among other adverse effects, clozapine-induced hypersalivation (CIH) occurs in approximately half of all patients [3, 4]. We report the case of a 30-year-old male with refractory schizophrenia; in this patient, the remission of residual positive symptoms, as well as the reduction of CIH, was achieved by treatment with clozapine augmented with amisulpride.

## 2. Case Report

The patient was admitted to the inpatient unit of a tertiary psychiatric hospital with command auditory hallucinations, persecutory delusions, and suicide ideation, despite regularly taking 900 mg/day clozapine and 30 mg/day aripiprazole. This patient was also being given 1 g/day valproic acid and 1.5 mg/day haloperidol twice daily; haloperidol was initiated at admission, in the emergency room. His first psychotic episode occurred at the age of 21, when he set himself on fire motivated by auditory hallucinations.

This patient was evaluated on September 25, 2014, and was found to have moderate positive symptoms using the Brief Psychiatric Rating Scale (BPRS; total score: 4; suspiciousness: 3; hallucinations: 0; and unusual thought content: 1); mild suicidal ideation according to Hamilton Depression Rating Scale (HDRS; 1 point for the third item, “feels life is not worth living”); and massive salivation using the Nocturnal Hypersalivation Rating Scale (NHRS; score: 4; “very severe—awakening due to hypersalivation”). The patient suffered from hypersalivation before treatment with aripiprazole was initiated, and treatment with aripiprazole, as well as the addition of haloperidol from the admission, did not worsen the CIH according to the patient, confirmed by information from his caregiver. Aripiprazole was gradually discontinued, and up to 50 mg/day amisulpride was added. Four weeks later, his score on the NHRS was 1 (“mild—signs of saliva on pillow”), and he had no positive symptoms according to the BPRS and no suicidal ideation. Ten weeks after discharge, his positive symptoms remained in remission, and he continued to have no suicide ideation. However, at this third assessment, sialorrhea was found to have worsened, although it was less severe than it had been at the first evaluation.

## 3. Discussion 

The efficacy of clozapine augmentation with amisulpride has been demonstrated in a single-blind, randomized study [5]. The efficacy of amisulpride may be related to its unique ability to blockade D_2_/D_3_ receptors, especially in limbic regions, rather than striatal structures [6]. Moreover, the D_2_ partial agonism of amisulpride helps to explain both its antipsychotic property and fewer incidences of extrapyramidal symptoms [7].

Few studies have reported the use of amisulpride for the control of CIH. Kreinin et al. (2006) found a considerable reduction in CIH following amisulpride augmentation, in addition to significant improvements in negative symptoms [8]. Different from clozapine, amisulpride does not display significant affinity for muscarinic receptors, whose activity probably is the main mechanism of excessive salivation [7, 9]. However, the antisalivation mechanisms of the interaction between clozapine and amisulpride remain unknown, and experimental studies have failed to demonstrate any inhibitory influence of amisulpride on the flow of saliva [10]. The positive association between higher doses of clozapine and intensity of the muscarinic activity may collaborate to the incidence of CIH in patients with schizophrenia and refractory symptoms [7].

We cannot discard the possibility of an eventual change in the pattern of adherence as the reason for the fluctuations in the severity of excessive salivation after the inpatient treatment. Still, as the prescription was the same since the discharge from the hospital, it is unlikely that the fluctuation in the severity of excessive salivation during the follow-up is due to changes in the pharmacological profile of the treatment.

Further studies are needed to clarify the mechanisms underlying both of these actions of this drug. To our knowledge, this is the first case report describing the benefits of clozapine augmentation with amisulpride for reducing both positive symptoms and CIH.
